# Update on new GH-IGF axis genetic defects

**DOI:** 10.20945/2359-3997000000191

**Published:** 2019-11-01

**Authors:** Gabriela A. Vasques, Nathalia L. M. Andrade, Fernanda A. Correa, Alexander A. L. Jorge

**Affiliations:** 1 Hospital das Clínicas Faculdade de Medicina Universidade de São Paulo São Paulo SP Brasil Unidade de Endocrinologia Genética, Laboratório de Endocrinologia Celular e Molecular (LIM25), Hospital das Clínicas, Faculdade de Medicina, Universidade de São Paulo, São Paulo, SP, Brasil; 2 Hospital das Clínicas Faculdade de Medicina Universidade de São Paulo São Paulo SP Brasil Unidade de Endocrinologia do Desenvolvimento, Laboratório de Hormônios e Genética Molecular (LIM42), Hospital das Clínicas, Faculdade de Medicina, Universidade de São Paulo, São Paulo, SP, Brasil

**Keywords:** Growth hormone, short stature, IGF, somatotropic axis

## Abstract

The somatotropic axis is the main hormonal regulator of growth. Growth hormone (GH), also known as somatotropin, and insulin-like growth factor 1 (IGF-1) are the key components of the somatotropic axis. This axis has been studied for a long time and the knowledge of how some molecules could promote or impair hormones production and action has been growing over the last decade. The enhancement of large-scale sequencing techniques has expanded the spectrum of known genes and several other candidate genes that could affect the GH-IGF1-bone pathway. To date, defects in more than forty genes were associated with an impairment of the somatotropic axis. These defects can affect from the secretion of GH to the bioavailability and action of IGF-1. Affected patients present a large heterogeneous group of conditions associated with growth retardation. In this review, we focus on the description of the GH-IGF axis genetic defects reported in the last decade. Arch Endocrinol Metab. 2019;63(6):608-17

## INTRODUCTION

**Figure 1:**
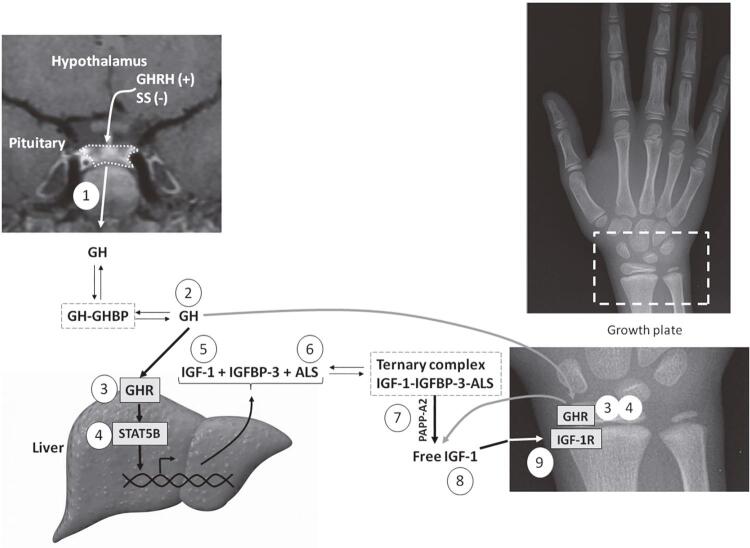
Schematic representation of the GH-IGF axis indicating the main disorders in this system: 1) growth hormone deficiency (GHD); 2) bioinactive GH; 3) GH insensitivity by GHR defects; 4) GH insensitivity by STAT5B defects; 5) IGF-1 deficiency; 6) acid-labile subunit (ALS) deficiency; 7) defects in the proteolytic cleavage of IGFBPs by PAPPA2 deficiency; 8) bioinactive IGF-1; and 9) IGF insensitivity caused by IGF1R defects.

The somatotropic axis is the main hormonal regulator of growth. Growth hormone (GH), also known as somatotropin, and insulin-like growth factor 1 (IGF-1) are the key components of the somatotropic axis. GH is secreted by somatotropes of the anterior pituitary gland, in a pulsatile manner, under stimulation of growth hormone-releasing hormone (GHRH), produced in the hypothalamus, and ghrelin (GHS), which is also secreted by the gastric cells. Somatostatin, another hypothalamic hormone, has an inhibitory action on GH secretion. GH acts by binding to its receptor (GHR), a homo-dimeric transmembrane receptor belonging to the citokine receptor superfamily. GH binding results in dimerization of the GHR and phosphorylation of JAK2, a tyrosine kinase associated with GHR. JAK2 phosphorylation leads to activation of different intracellular pathways, leading to direct metabolic effects or regulating the gene transcription, which may involve a family of signal transducers and activators of transcription (STATs). The most important STAT implicated in the growth-promoting signaling of GHR is STAT5B. This protein stimulates the synthesis of IGF-1 and of other molecules that increase IGF-1 bioavailability (Figure 1) (1,2).

The GH effect of promoting growth occurs mainly through IGF-1 production. Circulating IGF-1 is predominantly produced by the liver. Other tissues can produce IGF-1 that acts in a paracrine/autocrine manner, as occurs in the growth plate. Most of circulating IGF-1 is carried by a ternary complex, consisting of one molecule of IGF-1, one molecule of IGF binding protein type 3 (IGFBP-3) and one molecule of acid-labile subunit (ALS). This complex is essential to decrease the availability of free IGF-1 to the tissues, extending its serum half-life (Figure 1). IGF-1 is the main growth factor in postnatal growth. In the prenatal period, IGF-1 and IGF-2 have essential role in intrauterine development. IGF-1and IGF-2 act by binding to the cell surface tyrosine kinase receptor called IGF1R. This binding activates intracellular signaling, as the PI3K/AKT pathway, important for cell growth and proliferation (1,3).

**Figure 2:**
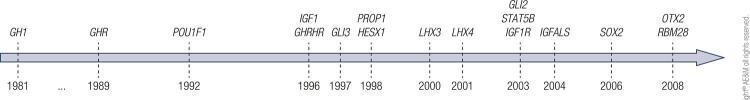
Historical timeline with genes previously associated with GH-IGF-1 axis defects until 2009.

Defects in many genes related to the somatotropic axis lead to a large heterogeneous group of conditions associated with growth retardation (4). Studies in this area are long-standing and, in 1929, researchers hypothesized that some genes would be responsible for pituitary development and function (5). After many reports of familial cases of isolated GH deficiency, deletions in *GH1* gene were recognized as its first monogenic cause in 1981 (6). Subsequently, in 1989, autosomal recessive mutations in *GHR* were established as cause of severe short stature with complete insensitivity to GH (7). A candidate gene for the hypopituitarism phenotype, named pituitary-specific positive transcription factor 1 (*POU1F1,* formally *PIT1*) was reported in 1990 based on a spontaneous dwarf mice (8). Two years later, an autosomal recessive mutation in *POU1F1* was identified in patients with GH, PRL and TSH deficiency (9). Also in 90s, inactivating mutations of the transcription factor of POU1F1 gene (*PROP1)* in an autosomal recessive pattern were also described in individuals with hypopituitarism, with or without gonadotropic deficiency (10). In the same period , homozygous loss-of-function mutations in *IGF-1* gene were reported in a child with intrauterine growth retardation, microcephaly, retarded intellectual development, and severe postnatal growth failure (11). Genes that were associated with GH-IGF-1 axis defects before 2009 are depicted in a historical timeline (Figure 2).

To date, defects in more than forty genes were associated with an impairment of the somatotropic axis. These defects can affect from the secretion of GH to the bioavailability and action of IGF-1. In this review, we describe the GH-IGF axis genetic defects reported in the last ten years. For didactic purpose, we divided this review in two main topics: “new genetic defects associated with GH deficiency” and “new genetic defects associated with GH-IGFs axis”.

## NEW GENETIC DEFECTS ASSOCIATED WITH GH DEFICIENCY

Growth hormone deficiency can be either isolated (IGHD) or combined with other pituitary hormones deficiencies (CPHD). It can be congenital or acquired and, regarding neuroimaging, it can present with no abnormalities or a wide range of structural changes of the hypothalamic-pituitary region. The pituitary stalk interruption syndrome (PSIS) has been classified as a distinct clinical picture characterized by hormone deficiencies, a thin, interrupted or absent pituitary stalk, usually associated with an ectopic posterior pituitary and hypoplasia of the anterior pituitary (12). Apart from the subtle midline malformation described in the PSIS, it is not unusual that patients with genetic defects present other complex phenotypes. The most common are: complex craniofacial malformations, septo-optic dysplasia (SOD) and developmental delay/intellectual disability (13). After the description of the classic and most frequent genetic defects associated with GH deficiency, many other genes have been associated with congenital hypopituitarism, most of them in a small subset of patients with complex phenotypes or PSIS. It has also been recently advocated that some patients could have more than one gene affected, which points to a digenic or oligogenic cause of GH deficiency. However, more studies are needed to explain the individual role of each mutation or its synergy in the pathogenesis of these phenotypes (14). Table 1 has a comprehensive list of these new genetic defects, and some of which will be discussed below.

**Table 1:** New genes associated with growth hormone deficiency either isolated (IGHD) or combined (CPHD) and their clinical features

Gene	Inheritance	Hormonal deficiencies	Anterior pituitary	Posterior pituitary	Other features	Reference
GLI2	Dominant	IGHD or CPHD	↓	Ectopic/NV	Polydactyly, HPE, craniofacial malformations	(16)
FGF8	Recessive	HH, IGHD or CPHD	↑ or NL	Topic	SOD, HPE, KS, Moebius syndrome	(18)
FGFR1	Dominant	HH or CPHD	NL or ↓	Ectopic or Topic	SOD, midline craniofacial malformations, corpus callosum abnormalities	(17)
PAX6	Dominant	IGHD or CPHD	↓	Topic	Midline craniofacial malformations, ophthalmologic abnormalities	(58)
GLI3	Dominant	IGHD or CPHD	A or ↓	Topic	Pallister-Hall syndrome	(59)
ARNT2	Recessive	CPHD	↓	Topic	Brain, eye, kidney and urinary tract abnormalities, corpus callosum abnormalities	(20)
CDON	Dominant	CPHD	A	Topic or Ectopic	HPE, maternal ethanol exposure worsens the phenotype	(22)
GPR161	Recessive	IGHD or CPHD	↓	Ectopic		(60)
IGSF1	X-linked	CPHD	NL	Topic	Macroorchidism, undetectable prolactin	(21)
PROKR2	Dominant or recessive	HH, IGHD or CPHD	NL or ↓	Topic or Ectopic	SOD, Hirschsprung disease	(17)
TGIF1	Dominant	CPHD	↓	Ectopic	HPE, midline craniofacial malformations	(61)
TCF7L1	Dominant	IGHD	↓	NV	SOD	(62)
PROK2	Dominant	CPHD	NL	Ectopic	Digenic	(14)

IGHD: Isolated growth hormone deficiency; CPHD: combined pituitary hormone deficiencies; HH: hypogonadotropic hypogonadism; A: aplastic; NL: normal; ↓: small; ↑: large; NV: non-visualized; SOD = septo optic dysplasia, HPE = holoprosencephaly, KS = kallmann syndrome

### GLI2 – a gene frequently involved in GH deficiency

GLI2 is a transcription factor that contains a zinc-finger region responsible for binding to DNA and is involved in the Sonic Hedgehog (Shh) signaling pathway, which is implicated in cellular proliferation during embryogenesis. It is important for hypothalamus, infundibulum and posterior pituitary lobe formation (15).

Regarding the clinical picture, patients with *GLI2* mutations may have IGHD or CPHD. An ectopic posterior pituitary is a frequent finding as well as polydactyly. In some patients, other midline craniofacial malformations, such as cleft palate and a median maxillary incisor, have been described. The presence of polydactyly should prompt to *GLI2* testing and is a marker of severity. There are also rare reports of associated diabetes insipidus. The inheritance is autosomal dominant with incomplete penetration and it is possible that other genetic and/or environmental factors may play a role in the phenotype. In a recent review of the important role of *GLI2* mutations in GH deficiency, among 284 patients in whom the complete coding region was sequenced, 15 patients (5%) had either loss-of-function or non-synonymous *GLI2* rare variants making it the most frequent genetic defect found in these patients, although one cannot confirm the pathogenicity of all of them (15,16).

### FGF8, FGFR1, PROKR2 and PROK2 – the overlap between GH deficiency and hypogonadotropic hypogonadism

The hypotheses that mutations in genes underling Kallmann syndrome could also lead to hypopituitarism was made based on the knowledge that an embryogenic structure called preplacodal gives rise to the adenohypophyseal, lens, and olfactory placodes. Therefore, defects in genes involved in the development of these structures could be responsible for both phenotypes (17). Until now, many genes previously associated with hypogonadotropic hypogonadism have also been associated with IGHD or CPHD with or without complex phenotypes. There is a great genetic heterogeneity with autosomal dominant or recessive inheritance with incomplete penetrance. Among the clinical pictures described, there are different combinations of pituitary hormonal deficiencies associated with SOD, HPE, PSIS and Moebius syndrome (17-19).

### ARNT2 and IGSF1 – GH deficiency with very unusual phenotypes

ARNT2 is a transcription factor member of the basic HLH-PAS subfamily proven to be important for hypothalamic development. Using exome sequencing, a mutation in an autosomal mode of inheritance was found in a family with an unusual and distinct combination of clinical manifestations in six affected children (OMIM 615926) (20). The patients had a variable combination of pituitary hormone deficiencies: ADH, ACTH, TSH, LH/FSH and GH. MRI of the brain revealed a similar pattern of abnormalities in all patients: non-visualized posterior pituitary, thin pituitary stalk, hypoplastic anterior pituitary, hypoplastic frontal and temporal lobes, thin corpus callosum and a global delay in brain myelination. All six children developed secondary microcephaly, severe global developmental delay and generalized tonic-clonic/partial seizures. Neurological examination revealed total body spastic cerebral palsy and minimal pupil response to light. All patients were dysmorphic and presented with additional features, such as gastro-oesophageal reflux and neurogenic bladder (20).

IGSF1 is a plasma membrane glycoprotein expressed in Rathke’s pouch and the anterior pituitary gland. Mutations in *IGSF1* are X-linked and the main clinical characteristics of male patients are congenital central hypothyroidism and macroorchidism (OMIM 300888). A variable proportion of patients presented partial and transient GH, prolactin deficiency, disharmonious pubertal development and increased BMI. In one patient with GH deficiency, MRI was abnormal with hypoplasia of the corpus callosum and small stalk lesion (PSIS) (21).

### CDON – GH deficiency and the link with environmental enhancers

CDON is a cell surface sonic hedgehog (SHH) binding protein that promotes SHH signaling activity. A heterozygous nonsense mutation in *CDON* was described in a patient with PSIS with perinatal complications: breech presentation, cesarean delivery, neonatal jaundice with increased conjugated bilirubin. The neuroimaging evaluation indicated small anterior pituitary, absent stalk, and ectopic posterior pituitary. The patients’ mother had the same mutation with a different phenotype – congenital convergent strabismus – which pointed to incomplete penetrance (22). Bae and cols. reported a broad spectrum of Holoprosencephaly (HPE) phenotypes in association with *CDON* mutations, including hepatic cholestasis and hypotelorism (OMIM 614226) (23). Incomplete penetrance has been described in many other families with pituitary anomalies with mutations involving other genes, and one mechanism proposed for this phenomenon is the gene-environment interactions (24). Regarding CDON, a synergy between a *Cdon* mutation in the mouse and ethanol leading to HPE was reported (25). This has also been observed with *Gli2*^*+/−*^ and *Shh*^*+/−*^ mice (26).

Regarding the new genetic defects reported in the last ten years causing GH deficiency (Table 1), we discussed the ones with an interesting clinical picture or etiopathogenic aspect. All the genes discussed above present a strong level of evidence as disease causing.

## NEW GENETIC DEFECTS ASSOCIATED WITH GH-IGFS AXIS

Although defects in *GHR*, *IGF1* and *IGF1R* genes have been associated to growth disorders for a long time, the number of described individuals with heterozygous mutations in these genes has increased recently, expanding the genotype-phenotype correlations. Additionally, new genetic defects downstream of GH were described (Table 2).

**Table 2:** New genetic defects of GH-IGF axis and their clinical features

Gene	Inheritance	Hormonal levels of GH/IGF-1/IGFBP-3	Clinical features	References
STAT5B	AD (with dominant-negative effect)	High/low/low	Partial GH insensitivity, mild eczema and elevated IgE	(35)
STAT3	AD (with gain of function)	High/low/low	Partial GH insensitivity and immune dysregulation	(37)
PAPPA2	AR	High	Mild microcephaly, small chins and long thin fingers; low free IGF-1	(44)
PIK3R1	AD	ND	Complex phenotype of SHORT syndrome	(46-48)
IGF2	ADp	Normal or slightly high	Phenotype similar to Silver-Russel syndrome; low IGF-2 levels	(50)

AD: autosomal dominant; AR: autosomal recessive; ADp: AD with paternal transmission; ND: no data.

### Update of genotype-phenotype correlations of GHR, IGF1 and IGF1R

Autosomal-recessive mutations in the GHR gene (*GHR)* cause a classical picture of GH insensitivity (GHI), characterized by extreme short stature, facial dysmorphic features and IGF-1 deficiency (OMIM 262500) (27). Several heterozygous variants in *GHR* have been associated with the short stature phenotype, although few have been proven as causal. Three patients with growth failure caused by heterozygous *GHR* mutations with a dominant-negative effect have been reported recently, totaling seven patients to date (28). These patients had a mild GHI phenotype which can be initially classified as idiopathic short stature (OMIM 604271). They usually presented detectable levels of IGF-1 and less severe short stature than individuals with classical GHI.

Homozygous defects in *IGF1* lead to pre and postnatal short stature, microcephaly and intellectual impairment (OMIM 608747). Since the first patient described, the researchers noted that carriers of heterozygous *IGF1* mutations had a reduced height and head circumference (11). In 2010, a heterozygous frameshift mutation in *IGF1* was associated with short stature and microcephaly in two siblings (29). The mother who carried the same mutation, which had been inherited by her father, presented a milder phenotype. This fact raised the hypothesis that the probands were more affected because they were born from a heterozygous mother, with a combination of fetal and maternal IGF-1 deficiency (29). Two years later, a heterozygous *IGF1* splice site mutation was identified in a boy with postnatal short stature (height SDS of -4.0) with low IGF-1 level (SDS of -2.2). Other four relatives with short stature carried the same mutation (30). In 2014, a complete heterozygous deletion in *IGF1* was identified in a child with short stature initially classified as idiopathic. The proband had a history of intrauterine growth restriction and was born with weight and length SDS of -1.5 and -1.2, respectively. He presented microcephaly and developmental delay (31).

While defects in *IGF1* are quite rare, many heterozygous defects in IGF1R gene (*IGF1R*) have been reported. Initially, studies described deletions encompassing *IGF1R* and, years later, point mutations were also identified in children with growth failure. According to a recent study, there are 36 different probably pathogenic variants in *IGF1R* (32)*.* Classically, patients with heterozygous defects in *IGF1R* present pre and postnatal growth retardation, microcephaly and IGF-1 levels above the mean for age and sex (OMIM 270450). Other clinical manifestations include mental and motor development delay, cardiac defects and dysmorphic features (triangular face, clinodactyly, pectus excavatum). The degree and the prevalence of the other clinical features appear to be higher in patients with *IGF1R* deletions than in those carrying point mutations. In addition, among those individuals with *IGF1R* deletions, the severity of the clinical condition appears to be dependent on the location of the breakpoints (32).

### Autosomal dominant defects in STAT5B

STAT5B acts as a key transcription activator of GH signaling. It has been known, since 2003, that homozygous mutations in *STAT5B* cause extreme short stature as part of a GH insensitivity syndrome associated with immune dysregulation (represented clinically by progressive pulmonary disease and eczema) (OMIM 245590) (33). First-degree relatives of index cases carrying *STAT5B* mutations were significantly shorter than individuals from the same population (height SDS of -1.4 ± 0.8 *vs.* -0.4 ± 0.8; *p* < 0.001) (34). Additionally, individuals who carry heterozygous mutations in *STAT5B* were reported to be shorter than their non-carrier family members (height SDS difference of -0.6, *p* = 0.009), although all of them had height within the normal range. Besides the growth impairment, eczema was observed in 4 of 32 carriers (34). Reconstitution experiments with loss-of-function mutations inherited by recessive manner showed a lack of expression of the mutant protein (35). Therefore, the slight height reduction observed in carriers of mutations in *STAT5B* could be explained by partial haploinsufficiency.

Recently, three heterozygous mutations in *STAT5B* were reported as cause of short stature with mild GH insensitivity in three children from different families (36). Functional evaluation revealed that those three mutations had dominant-negative effects. Mutated STAT5B proteins can be phosphorylated upon GH stimulation and can form dimers with themselves and with wild-type STAT5B proteins. In total, eleven individuals from the three families carried dominant-negative heterozygous *STAT5B* mutations. The degree of short stature had a high variability inter- and intra-familial and the height SDS of the probands ranged from -5.3 to -2.9. Although no one had severe immune dysregulation, the majority of affected individuals presented mild eczema (7 of 11) and elevated IgE (8 of 11) (36).

### Gain of function mutations in STAT3

STAT3 is a cytosolic protein that acts in many physiological processes stimulated by a wide variety of cytokines and growth factors, including inteleukin-6 (IL-6). Studies with cell cultures systems showed that STAT3, under IL-6 activation, induces an acute-phase response in hepatoma cells and stimulates proliferation in B lymphocytes (37). In 2014, Flanagan and cols. described that *de novo* germline activating *STAT3* mutations were a new monogenic cause of autoimmunity (OMIM 615952). The five affected individuals had early-onset (diagnosis at <5 years) autoimmune disease (e.g. type 1 diabetes, hypothyroidism, celiac disease) and all of them had short stature (38). Short stature was also observed in 7 of 13 patients with immune dysregulation and gain-of-function (GOF) mutations in *STAT3* (39). In another study, a boy presented height SDS of -2.6 at 5.5 years with IGF-1 of 37 µg/L (SDS of -2.2) (40).

We could speculate that the growth impairment observed in those patients could be due solely to their chronic disease. However, in 2018, Gutiérrez and cols. described two unrelated children with partial GH insensitivity and immune dysregulation caused by *de novo* GOF *STAT3* mutations (41). One patient had height SDS of -6.4 at 2.4 years of age, with elevated serum levels of GH and prolactin associated with undetectable levels of IGF-1, while the other, at the age of 3, had height SDS of -5.4. Both presented eczema, hypothyroidism, chronic diarrhea and recurrent infections. Functional evaluation using a STAT3-responsive dual-luciferase reporter assay showed an increased reporter activity of the two STAT3 mutants in comparison to wild-type STAT3. Although mutated STAT3 were not constitutively phosphorylated, they presented delayed dephosphorylation, leading to enhanced activity. Additionally, under unstimulated conditions and under GH treatment, both GOF *STAT3* variants decreased STAT5B transcriptional activity, suggesting a negative impact in the GH signaling pathway (41).

### Defects in the proteolytic cleavage of IGFBPs gene (PAPPA2)

IGF-1 can be found in the plasma bound to six IGFBPs. The ternary complex, composed by IGF-1, IGFBP-3 and ALS, is the form in which IGF-1 circulates with the highest half-life. However, to act on its receptor, IGF-1 must be released from the ternary complex (42,43). This task is done by the metalloproteinase pregnancy-associated plasma protein A2 (PAPPA^2^), a serum and tissue protease responsible for the specific proteolytic cleavage of IGFBP-3 and -5 (44).

Based on the study of two different families evaluated through whole exome sequencing, mutations in *PAPPA2* in an autosomal recessive inheritance were established as the cause of short stature with markedly elevated IGF-1 and IGFBP-3 concentrations (45). One family, with two affected children, harbored a novel homozygous frameshift mutation in *PAPPA2* (p.Asp643fs25*). In the other family, with three affected children, a novel missense variant (p.Ala1033Val) was observed. In vitro studies showed that both mutations led to absence of the proteolytic activity of the PAPPA2. Four of five patients had low free IGF-1 concentrations, despite the high levels of circulating IGF-1. Two children were born small for gestational age, while the others were born within the low normal range and smaller than the unaffected siblings. The growth impairment was becoming more prominent with age and other characteristics were observed, as small chins and long thin fingers (45,46). Three of five affected children presented microcephaly, with no history of neuropsychomotor development delay. Bone age was consistent with chronological age in all children (45).

### Defects in the regulatory subunit of PI3K gene (PIK3R1)

The phosphatidylinositol 3 kinase (PI^3^K) intracellular pathway is crucial to the action of IGF-1. After activation of tyrosine kinase receptor, substrates are phosphorylated and they bind to the regulatory subunit of PI3K, encoded by *PIK3R1* gene (47).

In 2013, heterozygous mutations in *PIK3R1* were established as the major cause of SHORT syndrome, a rare disease characterized by short stature, delayed dentition, characteristic facial features, ocular depression, lipodystrophy and hyperextensibility of joints (OMIM 269880). Several gene defects in *PIK3R1* were described (nucleotides insertion or deletion, nonsense and missense variants), with prevalence of *de novo* inheritance. One missense variant (p.Arg649Trp), within the context of a CpG motif, was recurrent in unrelated patients (47-49). There was a phenotype variability among the affected patients, including the degree of the growth impairment. Some individuals had prenatal short stature, with relative microcephaly, whereas others had height in the low-normal range. The mechanism of how mutations in *PIK3R1* result in the complex phenotype of SHORT syndrome is not completely understood. It is known that the regulatory subunit of PI3K has different isoforms expressed in several tissues (47-49).

### IGF2 defects

During the prenatal period, *IGF2* is expressed by the paternal allele in the placenta and has a fundamental role in promoting fetal growth. The clinical importance of the IGF-2 function was acknowledged from the study of patients with the Silver-Russell syndrome (SRS). Up to 60% of the cases of SRS are caused by the hypomethylation of the imprinted domain on chromosome 11p15.5, which leads to a decrease in the *IGF2* expression. The second most frequent cause of SRS is the maternal uniparental disomy of the chromosome 7. Children with SRS classically present prenatal onset short stature, relative macrocephaly, frontal bossing, triangular face, micrognathia, clinodactily, body asymmetry and feeding problems with low body mass index (50).

In 2015, Begemann and cols*.* described four individuals from the same family with SRS phenotype without a recognized molecular cause. Through exome analysis, the authors identified an *IGF2* nonsense mutation in all patients, inherited from their healthy fathers (OMIM 616489) (51). After this first report, other five studies identified variants in *IGF2* gene on the paternal allele causing SRS-like (52-56). Up to now, one missense and six loss-of-function variants (3 frameshift, 2 nonsense and 1 splice-site) in the *IGF2* gene were reported. All affected children were born small for gestational age regarding weight and length, had relative macrocephaly and short stature at preschool age (height SDS ranged from -3.3 to -6.2). They had varying degrees of feeding problems and neuropsychomotor developmental delay. Five probands presented cardiac abnormalities, including patent ductus arteriosus and atrial or ventricular septal defects. Male genital abnormalities, as hypospadia, penoscrotal transposition, cryptorchidism and ambiguous genitalia were also reported (51-53,56).

In addition to mutations in *IGF2*, Habib and cols. described new loss-of-function mutations in *HMGA2* and *PLAG1* genes in a cohort of suspected SRS patients. Both genes belong to the HMGA2–PLAG1–IGF2 pathway and act as positive upstream regulators of *IGF2* (54).

All genes described above have a good level of evidence regarding their implication on GH-IGF1 axis disruption. The group of the oldest genes (*GHR*, *IGF1* and *IGF1R*) in addition to *IGF2* have the highest level of evidence, as there are many reports of affected individuals from different families. Heterozygous mutations in *STAT5B* with a dominant-negative effect and homozygous mutations in *PAPPA2* also present a gene-disease clinical validity. Although defects in these genes were reported in only three and two different families, respectively, the variants segregated according to phenotype and had a convincing functional evaluation. Finally, *STAT3* and *PIK3R1* are also clinically relevant genes that impair the somatotropic axis. The phenotype of affected individuals are complex and there is a prevalence of *the novo* inheritance, reinforcing the gene-disease relationship.

## CONCLUSION

Although many phenotypes associated with the GH-IGF1 axis disruption have been recognized for many decades and the most frequent genetic defects were described before 2010, the discovery of defects in new genes has increased over the last decade. This increment was mainly due to the development of large-scale parallel sequencing techniques (targeted or exome sequencing), which allowed the analysis of many genetic defects simultaneously at low cost and in a shorter time than the previous candidate gene approach. Besides the identification of new genetic defects, this innovation permitted the expansion of the genotype-phenotype correlations related to known genes, boosting the clinical use of the genetic knowledge.

Since the GH-IGF1 axis is the main regulator of growth, it is insightful that defects at any step of this pathway could lead to short stature. These genes are responsible from the GH production to IGF-1 biodisponibility and action. In this review, we focused on the most relevant new genetic defects regarding the level of evidence recommended by the Clinical Genome Resource (ClinGen) (57).

As a future perspective, we speculate that defects in other genes which encoded intracellular signaling molecules could cause short stature. As these molecules are important to different pathways in different tissues, affected individuals would be rare and would present a multisystem phenotype. Also, we believe it will continue being a challenge to evaluate the clinical impact of variants in genes not previously associated to GH-IGF1 axis defects. This will demand careful and continuous research in this area.
